# Emergency medicine in Brazil: historical perspective, current status, and future challenges

**DOI:** 10.1186/s12245-021-00400-6

**Published:** 2021-12-22

**Authors:** Lucas Oliveira J. e Silva, Henrique Herpich, Henrique Alencastro Puls, Justin Guy Myers, Daniel Ujakow Correa Schubert, Ana Paula Freitas, Jule Santos, Marcus Vinicius Melo de Andrade, Hélio Penna Guimarães

**Affiliations:** 1 Associação Brasileira de Medicina de Emergência (ABRAMEDE), Porto Alegre, Brazil; 2 Department of Emergency Medicine, Mayo Clinic, Rochester, MN USA; 3 School of Medicine, Federal University of Health Sciences of Porto Alegre (UFCSPA), Porto Alegre, Rio Grande do Sul Brazil; 4 Department of Emergency Medicine, University of Michigan, Ann Arbor, MI USA; 5 Department of Emergency Medicine, University of North Carolina, Chapel Hill, NC USA; 6 Instituto D’Or de Pesquisa e Ensino-RJ, Rio de Janeiro, Rio de Janeiro Brazil; 7 Department of Emergency Medicine, Hospital de Pronto Socorro de Porto Alegre (HPS-POA), Porto Alegre, Rio Grande do Sul Brazil; 8 Department of Emergency Medicine, Hospital Regional de Santa Maria (HRSM), Brasília, Federal District Brazil; 9 Graduate Program in Health Sciences: Adult Health, Department of Internal Medicine, School of Medicine and Hospital das Clínicas, Universidade Federal de Minas Gerais, Belo Horizonte, Brazil; 10 Critical Patients Department, Albert Einstein Israelite Hospital, São Paulo, São Paulo Brazil

**Keywords:** Emergency medicine, Brazil, Acute care, Emergency care systems, International emergency medicine

## Abstract

**Background:**

Emergency medicine (EM) in Brazil has achieved critical steps toward its development in the last decades including its official recognition as a specialty in 2016. In this article, we worked in collaboration with the Brazilian Association of Emergency Medicine (ABRAMEDE) to describe three main aspects of EM in Brazil: (1) brief historical perspective; (2) current status; and (3) future challenges.

**Main text:**

In Brazil, the first EM residency program was created in 1996. Only 20 years later, the specialty was officially recognized by national regulatory bodies. Prior to recognition, there were only 2 residency programs. Since then, 52 new programs were initiated. Brazil has now 54 residency programs in 16 of the 27 federative units. As of December 2020, 192 physicians have been board certified as emergency physicians in Brazil. The shortage of formal EM-trained physicians is still significant and at this point it is not feasible to have all Brazilian emergency care units and EDs staffed only with formally trained emergency physicians. Three future challenges were identified including the recognition of EM specialists in the house of Medicine, the need of creating a reliable training curriculum despite highly heterogeneous emergency care practice across the country, and the importance of fostering the development of academic EM as a way to build a strong research agenda and therefore increase the knowledge about the epidemiology and organization of emergency care.

**Conclusion:**

Although EM in Brazil has accomplished key steps toward its development, there are several obstacles before it becomes a solid medical specialty. Its continuous development will depend on special attention to key challenges involving recognition, reliability, and research.

**Supplementary Information:**

The online version contains supplementary material available at 10.1186/s12245-021-00400-6.

## Background

Brazil is the largest country in Latin America. It is the fifth largest country in the world by total area and the sixth in population with approximately 210 million people [1]. Along with other low-to-middle income countries, Brazil suffers from public health problems such as high rates of infectious diseases and high mortality rates from sepsis [2, 3]. In similar fashion, homicides and traffic-related deaths account for almost two-thirds of all deaths from external causes, representing a significant threat to public health [4]. As violence remains a large unsolved issue, trauma imposes a large burden to the healthcare system.

Brazil experienced a period of economic and social progress starting in the early 2000s, when more than 29 million people left poverty and inequality declined significantly [5]. However, since 2015, the pace of poverty and inequality reduction has stagnated [5] and, as one of the most affected countries during the current coronavirus disease pandemic [6], Brazil will face several challenges in the near future. Given such scenario, the integrity and development of high-quality healthcare delivery will depend on the strength of front-line specialties such as emergency medicine (EM).

The country’s healthcare system is composed of both public and private apparatus, but most citizens are served by the Unified Health System (in Portuguese, “Sistema Único de Saúde” [SUS]) [7, 8]. This is a universal healthcare system that was implemented by the Brazilian Federal Constitution of 1988 and it has led to significant improvements in population health indicators [9, 10]. As commonly seen in other countries, emergency care units and emergency departments (ED) are a gateway into the healthcare system and the quality of its care will directly impact patients’ outcomes. Given the large size of Brazil and significant infrastructure disparities across the country, the evolution of EM as a structured specialty has been heterogeneous. However, there were recent positive developments. EM was recognized as an official specialty in 2016, which resulted in increased interest in the specialty as a career path and the opening of multiple training programs across the country [11].

Because of the recent changes, we believe there is a need for an updated report [12] in the current status of EM in Brazil. We worked in collaboration with the Brazilian Association of Emergency Medicine (in Portuguese, Associação Brasileira de Medicina de Emergência [ABRAMEDE]) to describe three main aspects of EM in Brazil: (1) brief historical perspective; (2) current status; and (3) future challenges.

## Brief historical perspective

### Recognition of emergency medicine as a specialty

Emergency medicine in Brazil has achieved critical steps toward its development in the last decades. Despite the differences and diversity of the healthcare system structure and access within the country, the lack of a trained professional to manage undifferentiated and critically ill patients has always been a widely recognized issue. In 1996, the first EM residency training program was created at the Hospital de Pronto Socorro (Porto Alegre, Rio Grande do Sul, Brazil) [12]. The program was initially not formally recognized by National regulatory bodies. Emergency medicine at that point was called “Urgency Medicine” and it was considered a subspecialty of Internal Medicine. The impact of having only one program in the whole country was yet very limited and localized to Southern Brazil.

More than 10 years later, in 2007, the first national EM conference was held in Gramado (Rio Grande do Sul, South of Brazil). During this conference, the “Letter of Gramado” was written. This document highlighted the potential pathways for the future of EM in Brazil. This was signed by influencing and respected professionals and then was sent to the main medical councils as well as to government representatives within the healthcare system. This letter was a milestone for the beginning of the movement toward official recognition as a medical specialty.

In 2008, ABRAMEDE was launched with the goals of representing Brazilian emergency physicians and fighting for the recognition of the specialty in the country, so that more residencies could be created, and official EM training could be instituted in medical schools’ curriculum. During that same year, the second residency training program started in Fortaleza (Ceará, Northeast of Brazil).

In 2010, another manifesto named “Letter of Porto Alegre” was published by a group of emergency physicians who wanted to highlight the weaknesses of the Brazilian emergency care system as well as request the urgent need for recognition of EM as a specialty (Appendix S1). Besides the recognition, this manifesto also claimed the need of having EM residency programs officially accredited and funded by the Brazilian government. Several national forums were held after the manifesto including the one led by the Brazilian Federal Council of Medicine (in Portuguese, “Conselho Federal de Medicina”) in 2011. This discussion occurred in the city of Brasilia (capital of Brazil) and set the specialty recognition as a major priority by National leadership. The Ministry of Health also provided important support as the residency spots were to be funded directly by the federal government. After several forums and debates to consolidate the view that the recognition would lead to improvements in the healthcare system, the proposal to officially create EM residency programs was approved in late 2015 by the Brazilian Federal Council of Medicine, the Brazilian National Council of Medical Residencies (in Portuguese, “Conselho Nacional de Residência Médica”), and the Brazilian Medical Association (in Portuguese, “Associação Médica Brasileira”). On August 2016, the official document recognizing EM as a medical specialty in Brazil was published [13]. Table 1 illustrates the several historical milestones before the recognition.

**Table 1:** Milestones of emergency medicine development in Brazil

**1992** ● Emergency Medicine course created as part of medical school curriculum of University of São Paulo (São Paulo, SP, Brazil)
**1996** ● First Emergency Medicine residency program—Hospital de Pronto Socorro (Porto Alegre, RS, Brazil)
**2003** ● Implementation of the National Emergency Care Policy and the creation of SAMU 192 as a national prehospital strategy.
**2007** ● First national conference of Emergency Medicine (Gramado, RS, Brazil) ● “Letter of Gramado” (Gramado, RS, Brazil)
**2008** ● Creation of the Brazilian Association of Emergency Medicine (ABRAMEDE) ● Second Emergency Medicine residency program—Hospital de Messejana (Fortaleza, CE, Brazil) ● Implementation of Emergency Care Units (in Portuguese, Unidades de Posto Atendimento [UPA])
**2009** ● Second national conference of Emergency Medicine (Fortaleza, CE, Brazil).
**2010** ● “Letter of Porto Alegre” (Porto Alegre, RS, Brazil) ● First national political forum to discuss emergency care in Brasília, DF (capital of Brazil)
**2011** ● Second national political forum to discuss emergency care in Brasília, DF (capital of Brazil). The need for the recognition of the specialty is acknowledged as a key priority. ● Third national conference of Emergency Medicine (São Paulo, SP, Brazil)
**2012** ● Third national political forum to discuss emergency care in Brasília, DF (capital of Brazil)
**2013** ● Fourth national conference of Emergency Medicine (Curitiba, PR, Brazil)
**2015** ● Emergency medicine approved to become an official medical specialty by the key national regulatory bodies.
**2016** ● Official recognition of Emergency Medicine as a specialty (on August 2016 the official document is released by the Federal Council of Medicine). ● Several new Emergency Medicine residency programs were launched nationally.

## Current status

### Do medical schools teach emergency medicine in Brazil?

Medical education in Brazil follows the European model and after the completion of high school, students attend medical school for 6 years. As part of medical school, all programs include a mandatory 2-year internship before graduation. After receiving the medical degree, graduates may apply for residency.

In 2019, Brazil had 342 registered medical schools, including both private and public schools. Based on the available data, EM teaching at medical school level appears to be suboptimal, allowing students’ sparse contact with this specialty during their training. The Brazilian Association of Medical Education has received funding from the Pan American Health Organization to study the impact of national curriculum guidelines on medical school curricula [14]. Within this project, they have developed an initiative to specifically understand the teaching of EM in Brazil. Several themes have emerged from these studies including schools without any curriculum in EM, teaching hospitals without an ED, students working in emergency care units without supervision, lack of EM specialists or other staff capable of teaching EM, and lack of a longitudinal plan where students are exposed to the topics from year 1 through year 6 of medical school [14, 15].

The most recent national guideline from the Ministry of Education (2014) recommends that 30% of the hands-on training during the last 2 years of medical school (i.e., internships) should be held in settings such as the public primary care clinics or emergency care units [15]. Although this recommendation mandates students to rotate in emergency care units or EDs during their internships, exposure to EM is yet irregular and the specialty itself is not widely known within medical schools. Most of the popularity of EM comes from EM interest groups (in Portuguese, “Ligas Acadêmicas”) where students create a parallel curriculum as a strategy to compensate for this educational weakness [16].

### Emergency medicine residency training

**Fig. 1:**
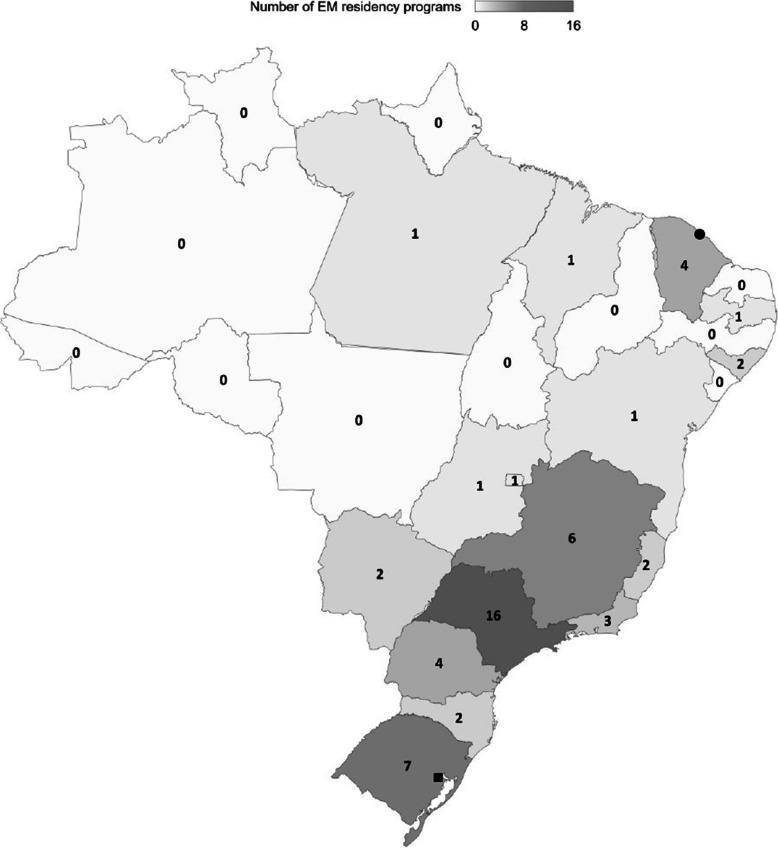
Geographical distribution of EM residency programs in Brazil in 2021. The square represents the location of the first EM residency program launched in Brazil. The circle represents the location of the second EM residency program launched in Brazil

As previously mentioned, the first Brazilian EM specialty program began in Porto Alegre, Rio Grande do Sul, in 1996. Its initial structure was of a 2-year program with 2 and later 4 spots per year, and, most recently, it changed to a 3-year duration program with 6 spots per year. Currently, all programs in the country have a 3-year curriculum. Before the recognition of the specialty in late 2015, there were only two programs available. Since then, 52 new programs have been listed by the Ministry of Education, totaling 54 programs across 16 federative units. The majority of programs are located in the South and Southeast of Brazil, with almost no programs in the Center-West and North of the country (Fig. 1). As of November 2021, there were 225 residency spots available per year for physicians interested in pursuing formal EM training (Appendix S2).

There is no unified residency application system for medical school graduates in Brazil and every hospital is responsible for structuring their own selection process. Some clusters of hospitals, however, have created unified processes in which medical students take one exam and apply to several different hospitals and programs. In 2019, some EM residency programs had up to 22 candidates per residency spot (data not published). In the Brazilian medical education system, this level of competitiveness is similar to the level experienced by applicants to other classic specialties such as Pediatrics, for example. This highlights how quickly the specialty got the attention of medical students who are now becoming more interested in pursuing a career in EM.

The educational curriculum across residency programs is still very heterogeneous [11]. In a survey of 35 programs, only 5 rotations were mandatory in all programs, including rotations in the ED intermediate acuity unit (“yellow” room), ED high acuity unit (“red” room), ICU, obstetrics and gynecology, and trauma [11]. The North American model is often followed [17], and national guidelines for a competency-based curriculum are being developed by ABRAMEDE leaders. An important aspect of current EM training in Brazil is the predominant focus on skills related to the management of critically ill patients. The high volume of critically ill patients being taken care of by emergency physicians is related to multiple factors including inadequate primary preventive care and lack of hospital and ICU beds [18, 19]. The lack of hospital and ICU beds not only contributes to ED crowding [20], but also leads to prolonged boarding times where patients stay in the ED for days while waiting for a transfer. Even in settings with adequate bed support, ED crowding can still be an issue [20]. For these reasons, the skill of coordinating crowding and optimizing ED patient flow is invaluable for Brazilian emergency physicians in training.

### Who practices Emergency medicine in Brazil?

Due to the late recognition of EM as a specialty, EDs are mostly staffed by physicians without formal training in EM. This includes recent medical graduates and physicians with other specialty training (e.g., Internal Medicine, General Surgery). A significant proportion of physicians work in the ED as a way to increase their monthly income while they are specializing in another area [21]. The vision of working in the ED as a temporary job may contribute to make the specialty of EM less attractive. The shortage of formal EM-trained physicians is still significant and at this point it is not feasible to have all Brazilian emergency care units and EDs staffed only with formally trained emergency physicians.

Board certification is being led by ABRAMEDE. In 2017, the first EM board exam occurred and physicians who have been practicing EM for years had the chance to get their official board certification. As of December 2020, 192 physicians [22] have been board certified as emergency physicians in Brazil, and, given the recent spike of new residency programs [11], the expectation is that this number will rapidly increase in the upcoming years.

Emergency physicians trained at the few formal residency programs available are now leading the way, and several have become chairs of EDs, taking important administrative positions. Besides working clinically and teaching at residency programs and universities, important roles include evaluation of appropriate ED patient flow, crowding, and the implementation of evidence-based protocols using EM-tailored clinical research. This scenario is similar to what occurred in the USA in the early 1980s [17].

### Emergency care at the pre-hospital setting

Pre-hospital care in Brazil has significant influence from the French model, although it has increasingly acquired its own characteristics. Brazil has ambulance services that use mixed strategies such as the “stay and play” or the “scoop and run.” Both of these approaches are provided by the SAMU (in Portuguese, “Serviço de Atendimento Móvel de Urgência”), which is public and widely available across the country. Private ambulance services are also available. Since 2003, SAMU has been chosen by the government as the standard model for emergency medical services (EMS) [23]. Despite its official national regulations in 2003, there have been reports of regional implementation of SAMU since the 1990s in some Brazilian cities. In 2017, SAMU covered approximately 79% of the Brazilian population [24]. However, its implementation is still unequal across states and regions, with some places fully covered and others with less than 50% coverage [25].

Currently, SAMU operates on a system where citizens may call the toll-free number 192 (same number in any part of the country) and ask for an ambulance. A physician in charge of medical control (unique characteristic of the system) will then decide about dispatching either a basic or an advanced life support unit. A basic ambulance unit will include a driver and a nurse technician. An advanced ambulance unit must include a driver, a physician, and a registered nurse. Some Brazilian states have also adopted a model of ambulance care where the Military Fire Department personnel trained as emergency medical technicians provide pre-hospital care, mostly to trauma patients [26]. For this reason, states such as Rio de Janeiro, for example, relies on both the Fire Department and SAMU for its public pre-hospital care. In contrast to several other countries, Brazil does not follow the concept of “paramedics” but rather have trained nurse technicians and registered nurses occupying this role.

Although certain certifications such as the *Pre-Hospital Trauma Life Support* course are required in order to work in the pre-hospital setting, there are no formal curricula and there is a low degree of adhesion to continuous educational programs among SAMU medical professionals [27]. As for the role of emergency physicians in this setting, ABRAMEDE currently recommends at least one pre-hospital rotation for EM residents during each of the 3 years of residency, taking into consideration the fact that Brazil remains relying on the presence of physicians in ambulances (advanced units).

Despite progress, pre-hospital EMS are generally under resourced, understaffed, and poorly equipped [26]. These challenges may be the reflection of the late recognition of EM as a specialty. It is possible that with the growth of the specialty and the incorporation of prehospital medicine as a subspecialty of EM, the quality of this service gradually improves.

### Emergency care units and emergency departments

There are essentially two types of acute care delivery facilities in Brazil: (1) emergency care units (in Portuguese, “Unidades de Pronto Atendimento” [UPAs]) and EDs. Both are available through the public and private systems, but most emergency care units are part of the SUS (public healthcare system).

In the public system, Brazil had 614 emergency care units (UPAs) spread across the national territory in 2018 [28]. They are classified in three different sizes, according to the population covered, the physical area, the number of available beds, and the capacity to care. These units are defined as facilities of intermediate complexity, between the primary care setting and hospital EDs. The UPA offers a 24/7 simple structure, with ultrasound, X-rays, electrocardiography, basic laboratory, and observation beds. Computerized tomography (CT) or more advanced imaging modalities are not available at these units. If more complex care is needed, patients are then transferred to referral hospitals.

The main purpose of these emergency care units is to provide accessible and rapid acute care in order to avoid the crowding of EDs at large referral hospitals. Although the system is geographically organized to build a comprehensive emergency care network [26], transfers are often difficult due to several issues such as lack of ambulances and lack of beds available at the referral hospital [29]. Importantly, in 2016, there was an estimate of 136 UPAs that were physically built but not properly functioning [29].

Besides resource constraints, one of the biggest challenges is that most UPAs are staffed by physicians who have never had any type of formal EM training. This is an opportunity for future tele-medicine initiatives where trained emergency physicians could support UPAs that are not adequately staffed or are at very distant locations from referral centers. Physicians often “moonlight” in these emergency care units to supplement their income while building their practices and working other jobs. Besides that, there is a high physician turnover and a predominance of recent medical graduates with limited experience [21].

As for EDs at large referral hospitals, there is a highly heterogeneous physician workforce. Some teaching hospitals where EM residency programs are held may have formally trained emergency physicians as part of the ED faculty; however, there is still a significant proportion of staff who have had other types of training such as Internal Medicine or General Surgery. Herpich and colleagues showed that the estimate proportion of faculty with prior EM residency or board certification in EM was less than 20% in 74% of surveyed EM residency programs [11]. The role of the emergency physician is not as clear as it is in other countries. In some EDs, the emergency physician is not responsible for seeing certain chief complaints. For example, patients with gynecological chief complaints may be seen directly by a gynecologist. Patients who come in with abdominal pain may be seen directly by a surgeon. Patients with orthopedic complaints may be seen directly by an orthopedic surgeon.

## Future challenges (the 3 R’s—recognition, reliability, research)

### Recognition

The recognition that a formally trained emergency physician is able to assess undifferentiated complaints when patients come to the ED is a critical step that will need to occur in the development of EM in Brazil. Using trained emergency physicians will keep other specialists ready for cases with higher complexity, the operating room, and regionalization of care. In other words, this would allow physicians from traditional specialties to focus on relevant cases that have been screened by front-line-trained EM providers.

Besides the recognition by our peers from other medical specialties, another key challenge will be to clarify the wrong idea that EM is necessarily associated with high working hours, working under resource constraints, low salaries, and stressful shifts. This will require active engagement with medical students to explain the importance of the specialty within the healthcare system and the increased appreciation of the emergency physician by healthcare system stakeholders.

### Reliability

While a standardized national residency curriculum is being developed by the leadership of ABRAMEDE, its implementation across all residency programs will be challenging, which may threaten the reliability of training across the country. As a large nation with highly heterogeneous emergency care practice, it is difficult to standardize training that fits the need of all regions. For example, some areas of the country may need emergency physicians taking care of critically ill patients only, working almost entirely as EM intensivists. Besides the challenge of organizing a curriculum that meets the true needs of our healthcare system, residents are frequently staffed and supervised by physicians from other specialties, which also makes the standardization of the training more difficult.

### Research

The development of academic EM in Brazil is another important step toward its recognition among other medical specialties. The country has gone through an increase of government-based investment in science and technology in the early 2000s, which resulted in an increase of scientific production from 10,521 articles in 2000 to 33,100 in 2009 [30]. However, most recently, Brazil has been facing a stagnant economy and significant decreases in research investment [31]. Despite economic issues, the EM community in Brazil needs to find ways to build a strong research agenda in order to increase the knowledge about the epidemiology and organization of emergency care. Most recently, ABRAMEDE launched its scientific journal called “Jornal Brasileiro de Medicina de Emergência (JBMEDE)” [11], which now allows academic emergency physicians to communicate their findings with the Brazilian EM community. The creation of this journal is a giant milestone toward the direction of EM being recognized in the house of Medicine not only for its existence but also for its scientific contributions.

As another way to foster EM research, a reasonable strategy could be the one taken by the critical care community in Brazil. Intensive Care Medicine was recognized as a distinct specialty in 2002 by the Brazilian Medical Association, a process led by the Brazilian Intensive Care Medicine Association (in Portuguese, “Associação de Medicina Intensiva Brasileira”). Five years later, the critical care community launched the Brazilian Research in Intensive Care Network, which is an active and independent organization. Since then, they have been able to endorse and run several multicenter observational and randomized controlled trials as well as support local and international studies and investigators [32]. The creation of this large research network did not only strengthen the recognition of the specialty in the country within other medical specialties but it also allowed intensivists to use local high-quality research data to make better decisions to their critically ill patients. Given the significant collaboration between the specialties of intensive care medicine and emergency medicine in Brazil, the EM community may be able to take lessons from their intensivists colleagues and build the EM research agenda through a similar pathway.

## Conclusions

Emergency medicine in Brazil has accomplished key steps in the last two decades but is yet to face several obstacles before it becomes a solid medical specialty. Its continuous development will depend on special attention to key challenges involving recognition, reliability, and research.

## Supplementary Information

Additional file 1: **Appendix S1.** First page of the Letter of Porto Alegre (In Portuguese). **Appendix S2.** Residency programs by Brazilian states and number of spots available.

## Data Availability

Not applicable.

## Abbreviations

ABRAMEDE: Associação Brasileira de Medicina de Emergência
ED: Emergency department
EM: Emergency medicine
EMS: Emergency medical services
ICU: Intensive care unit
SAMU: Serviço de Atendimento Móvel de Urgência
SUS: Sistema Único de Saúde
