# Supplementary material for: Emergency medicine in Brazil: historical perspective, current status, and future challenges
Source: Int J Emerg Med. 2021 Dec 22;14:79. doi: 10.1186/s12245-021-00400-6 (PMC8693143; doi:10.1186/s12245-021-00400-6)
Supplement: Supplementary file 1 — Appendix S1. First page of the Letter of Porto Alegre (In Portuguese). Appendix S2. Residency programs by Brazilian states and number of spots available. [file 12245_2021_400_MOESM1_ESM.docx]

**SUPPLEMENTARY MATERIAL**

**Appendix S1.** First page of the Letter of Porto Alegre (In Portuguese).

**Appendix S2.** Residency programs by Brazilian states and number of spots available.

| State | Program | Spots available | | |
| --- | --- | --- | --- | --- |
|  |  | R1 | R2 | R3 |
| AL | Hospital Geral do Estado Dr. Osvaldo Brandão Vilela | 4 | 4 | 4 |
|  | Unidade de Emergencia Dr Daniel Houly | 1 | 1 | 1 |
| BA | Secretaria Municipal Da Saúde | 6 | 6 | 6 |
| CE | Escola de Saúde Pública do Ceará | 6 | 6 | 6 |
|  | Instituto Dr José Frota – IJF | 4 | 4 | 4 |
|  | Hospital Regional do Cariri | 5 | 5 | 5 |
|  | Hospital Regional do Cariri | 5 | 5 | 5 |
| DF | Secretaria de Saúde Distrito Federal ESCS | 6 | 6 | 6 |
| ES | Faculdade Brasileira | 2 | 2 | 2 |
|  | Hospital Metropolitano S/A | 2 | 2 | 2 |
| MA | Hospital Esperança AS | 2 | 2 | 2 |
| MG | Hospital das Clínicas da UFMG | 10 | 10 | 10 |
|  | Irmandade de Nossa Senhora das Merces de Montes Claros | 6 | 6 | 6 |
|  | Hospital João XXIII – FHEMIG | 8 | 8 | 8 |
|  | Hospital Marcio Cunha - Fundação São Francisco Xavier | 2 | 2 | 2 |
|  | Hospital Metropolitano Odilon Behrens | 4 | 4 | 4 |
|  | Hospital Maternidade E Pronto Socorro Santa Lucia LTDA | 2 | 2 | 2 |
| MS | Associação Beneficente de Campo Grande | 3 | 3 | 3 |
|  | Hospital Regional do Mato Grosso do Sul Rosa Pedrossian | 3 | 3 | 3 |
| PA | SESMA - Secretaria Municipal de Saúde e Meio-Ambiente / Hospital de Pronto Socorro Municipal MP | 3 | 3 | 3 |
| PB | Hospital Nossa Senhora Das Neves S/A | 6 | 6 | 6 |
| PR | Hospital da Cruz Vermelha | 2 | 2 | 2 |
|  | Hospital Universitário Cajuru | 2 | 2 | 2 |
|  | Hospital Universitário do Oeste do Paraná | 2 | 2 | 2 |
|  | Curitiba Prefeitura Municipal | 2 | 2 | 2 |
| RJ | Instituto D'or de Pesquisa e Ensino | 2 | 2 | 2 |
|  | Hospital Geral de Nova Iguaçu | 2 | 2 | 2 |
|  | Hospital Municipal Miguel Couto | 2 | 2 | 2 |
| RS | Hospital das Clínicas de Porto Alegre | 6 | 6 | 6 |
|  | Hospital de Pronto Socorro de Porto Alegre | 6 | 6 | 6 |
|  | Hospital Nossa Senhora da Conceição | 6 | 6 | 6 |
|  | Hospital São Lucas da PUCRS | 4 | 4 | 4 |
|  | Hospital Universitário da UFSM | 4 | 4 | 4 |
|  | Universidade Luterana do Brasil (ULBRA) | 1 | 1 | 1 |
|  | Hospital Moinhos de Vento | 2 | 2 | 2 |
| SC | Fundo Municipal de Saúde De Florianópolis | 4 | 4 | 4 |
|  | Hospital Regional Hans Dieter Schmidt | 4 | 4 | 4 |
| SP | Faculdade de Ciências Médicas da Unicamp | 4 | 4 | 4 |
|  | Faculdade de Medicina da USP | 24 | 24 | 24 |
|  | Fundação Leonor de Barros Camargo | 4 | 4 | 4 |
|  | Hospital Alemão Oswaldo Cruz | 3 | 3 | 3 |
|  | Hospital das Clínicas da Faculdade de Medicina de Ribeirão Preto USP | 4 | 4 | 4 |
|  | Hospital Geral do Grajaú | 2 | 2 | 2 |
|  | Hospital Israelita Albert Einstein | 2 | 2 | 2 |
|  | Hospital Regional de Presidente Prudente | 2 | 2 | 2 |
|  | Hospital Santa Marcelina | 6 | 6 | 6 |
|  | Irmandade da Santa Casa da Misericórdia de Santos | 2 | 2 | 2 |
|  | Santa Casa de Misericórdia de Barretos | 6 | 6 | 6 |
|  | Faculdade de Medicina de São Jose do Rio Preto | 4 | 4 | 4 |
|  | Hospital São Lucas SA | 2 | 2 | 2 |
|  | Municipio de São Jose do Rio Preto | 4 | 4 | 4 |
|  | Irmandade Santa Casa de Misericórdia de São Paulo | 3 | 3 | 3 |
|  | Universidade Federal de São Paulo – Unifesp | 6 | 6 | 6 |

**Source:** Conselho Nacional de Residência Médica. Disponibilizado em 2021.
